# Endoscopic Removal of a Nitinol Mesh Stent from the Ureteropelvic Junction after 15 Years

**DOI:** 10.1155/2015/273614

**Published:** 2015-12-01

**Authors:** Tomaž Smrkolj, Domagoj Šalinović

**Affiliations:** ^1^Department of Urology, University Medical Centre Ljubljana, 1000 Ljubljana, Slovenia; ^2^Clinical Institute of Radiology, University Medical Centre Ljubljana, 1000 Ljubljana, Slovenia

## Abstract

We report a rare case of a patient with a large stone encrusted on a nitinol mesh stent in the ureteropelvic junction. The stent was inserted in the year 2000 after failure of two pyeloplasty procedures performed due to symptomatic ureteropelvic junction stenosis. By combining minimally invasive urinary stone therapies—extracorporeal shock wave lithotripsy, semirigid ureterorenoscopy with laser lithotripsy, and percutaneous nephrolithotomy—it was possible to completely remove the encrusted stone and nitinol mesh stent that was implanted for 15 years, rendering the patient symptom and obstruction free.

## 1. Introduction

Ureteropelvic junction (UPJ) obstruction is a functional or anatomic obstruction to urine flow from the renal pelvis to the ureter that, if left untreated, results in symptoms or renal damage. Most patients with UPJ obstruction are diagnosed in the perinatal period by widespread use of antenatal ultrasound; however, diagnosis is sometimes delayed until adulthood, when lumbar pain, infection, stones, and hematuria might occur [1]. UPJ obstruction was traditionally repaired surgically by open pyeloplasty [2]. With the advent of percutaneous and retrograde endoscopic techniques, endopyelotomy with an anterograde or retrograde approach has become an accepted minimally invasive alternative [3]. Although the success rate of the open surgical approach is reported to be 72%–98% [4], up to one-quarter of patients require at least one additional intervention. For rare patients, where multiple surgical attempts have failed and symptoms of UPJ obstruction persist, a viable alternative is double-J catheter placement with replacement at regular time intervals. In the 1990s, permanent implantable metallic mesh stents were introduced to the treatment of ureteric stenosis [5], which promised to offer a permanent solution; however, their use in patients with expected long-term survival was largerly abandoned due to very high rate of stone incrustations and difficulty of their removal when complications emerged.

## 2. Case Presentation

**Figure 1:**
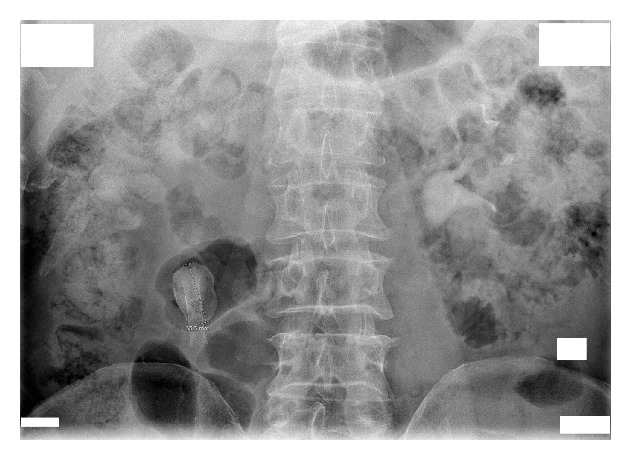
Intravenous urography shows a 35 mm stone encrusted on a nitinol mesh stent positioned in the right ureteropelvic (UP) junction, with delayed contrast medium excretion in the right kidney.

A man aged 65 years presented to the outpatient clinic in May 2014 with right-sided hydronephrosis and a large 35 mm stone encrusted on a nitinol mesh stent positioned in the UPJ. Intravenous urography confirmed delayed excretion of contrast medium in the right kidney (Figure 1), and the patient was admitted to the department of urology.

A right-sided open Anderson-Hynes pyeloplasty had been performed in 1991 due to symptomatic UPJ stenosis with symmetric renal function. Due to UPJ restenosis, open Culp-De Weerd pyeloplasty was done in 1999; however, in less than 1 year, UPJ stenosis recurred and necessitated double-J catheter placement into the right ureter, resulting in troublesome dysuria with lumbar and testicular pain. As an alternative to the double-J catheter, the patient was offered a nitinol mesh stent as a permanent solution, and the procedure was done by the interventional radiologist deploying a 4 cm long mesh stent to the UPJ in 2000. The patient was asymptomatic and lost to follow-up until 2006, when stone incrustations were identified on the mesh stent, although a MAG3 renal scan showed nearly symmetric renal function (right kidney 47%, left kidney 53%). One year later, increasing blunt right kidney pain emerged over months, and unsuccessful removal of the encrusted mesh stent was attempted using a percutaneous technique. Consequently, the patient agreed to placement of an indwelling large-diameter double-J catheter (Ch9) with subsequent yearly replacement using combined endoscopic and percutaneous techniques. The double-J catheter was permanently removed in 2011 due to recurring urinary tract infections and dysuria. Subsequent MAG3 renal scans in the following 2 years still showed nearly symmetric renal function (right kidney 47% and left kidney 53%) without obstruction.

**Figure 2:**
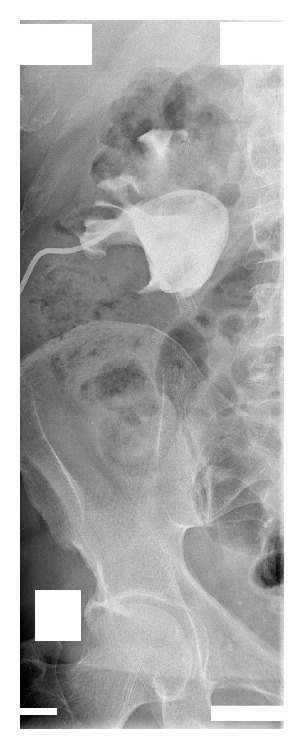
Right-sided nephrotomography shows complete obstruction at the encrusted nitinol mesh stent.

**Figure 3:**
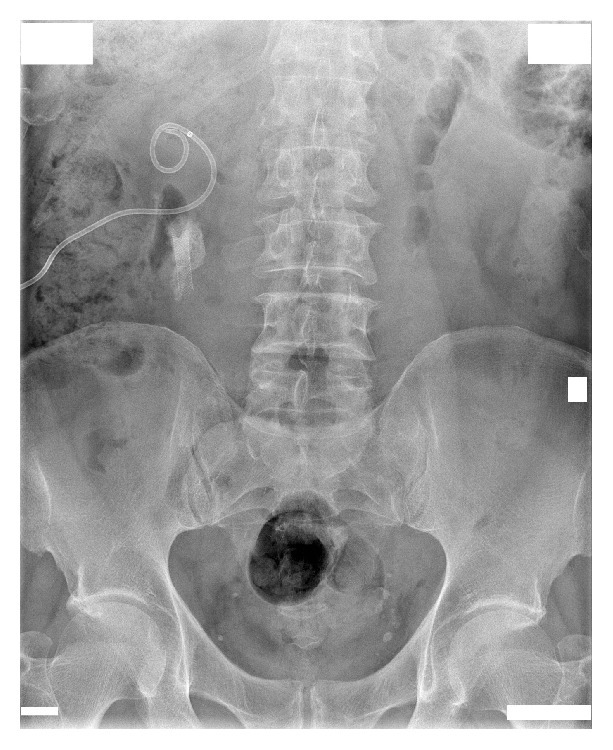
Plain X-ray image of the urinary tract after eight extracorporeal shock wave lithotripsy sessions shows partial disintegration of the stone in the right renal pelvis.

**Figure 4:**
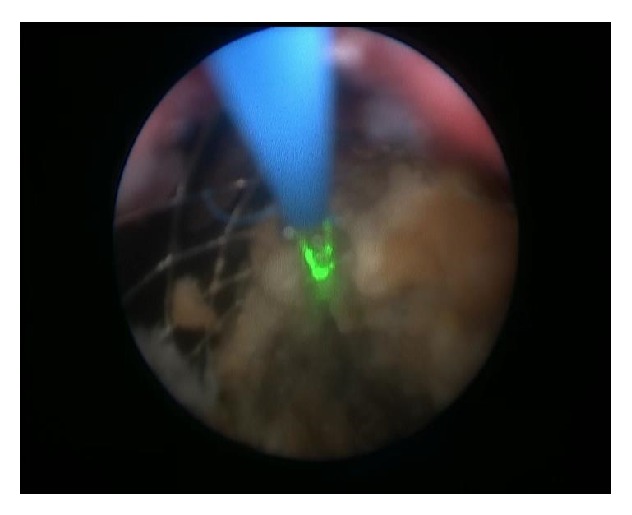
Image from the semirigid ureterorenoscopy with laser lithotripsy procedure.

**Figure 5:**
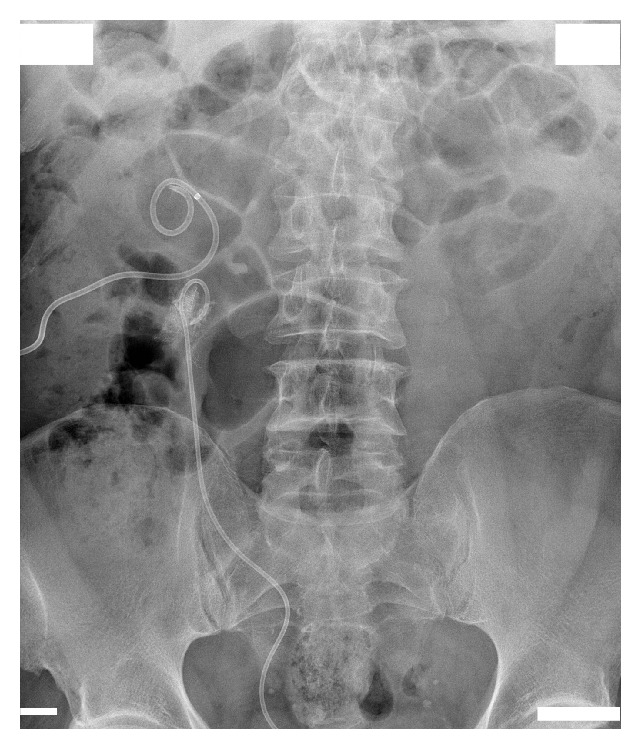
Plain X-ray image of the urinary tract after the semirigid ureterorenoscopy procedure shows that almost half of the mesh stent and its incrustations were completely disintegrated by laser energy.

**Figure 6:**
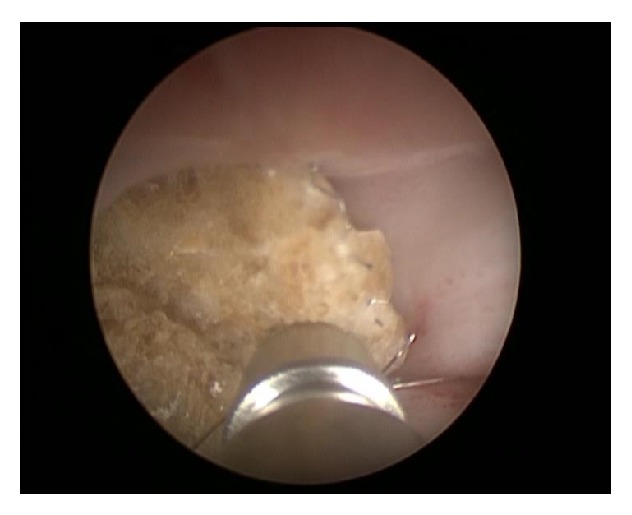
Image from the percutaneous nephrolithotomy shows grasper removing parts of the encrusted mesh stent.

**Figure 7:**
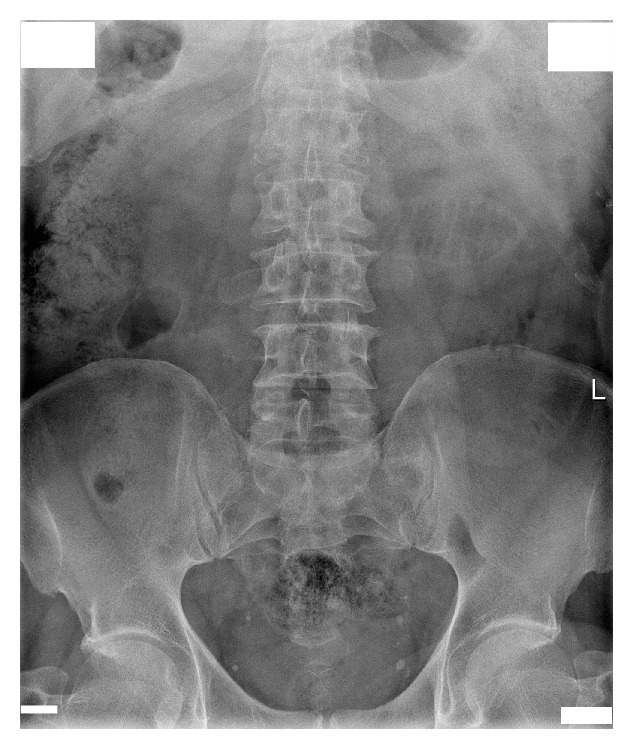
Plain X-ray image shows complete removal of the nitinol mesh stent and incrustations after percutaneous nephrolithotomy.

**Figure 8:**
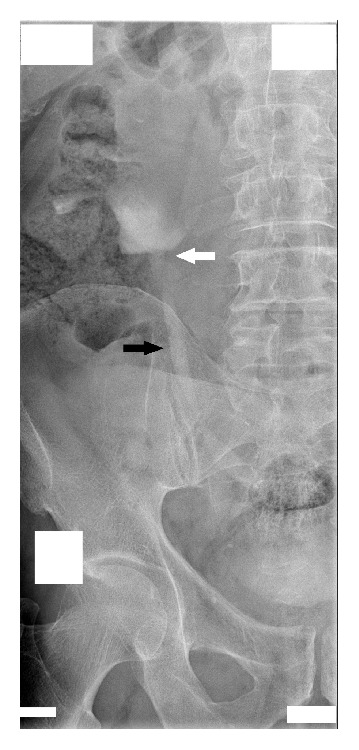
Intravenous urography 5 months after the last procedure shows chronically enlarged renal pelvis and a strong jet of contrast medium through the ureteropelvic junction (white arrow) and proximal and middle ureter (black arrow).

After admission in May 2014, a percutaneous nephrostomy tube was inserted into the right kidney, and complete obstruction was identified at the encrusted nitinol mesh stent (Figure 2). Eight extracorporeal shock wave lithotripsy (ESWL) sessions were performed with partial disintegration of the stone in the right renal pelvis (Figure 3); however, obstruction inside the mesh stent persisted. In October 2014, a semirigid ureterorenoscopy (URS) with holmium, yttrium-aluminum-garnet laser lithotripsy, was attempted, during which the nitinol mesh stent with the encrusted stone was dislodged from the UPJ, and almost half of the mesh stent and its incrustations were completely disintegrated by laser energy (Figures 4 and 5 and supplementary video file in Supplementary Material available online at http://dx.doi.org/10.1155/2015/273614). The remainder was removed during percutaneous nephrolithotomy (PCNL) in February 2015 (Figure 6 and supplementary video file), with a plain X-ray image on follow-up showing complete removal of the nitinol mesh stent and incrustations (Figure 7). Intravenous urography identified a chronically enlarged renal pelvis; however, calyces were only mildly dilated, and a strong jet of contrast medium was present through the UPJ and proximal and middle ureter (Figure 8). A MAG3 renal scan obtained 5 months after the last procedure showed nearly symmetric renal function (right kidney 45% and left kidney 55%) without obstruction, and the patient remained symptom-free.

## 3. Discussion

Endoluminal implantation of metal stents in the coronary arteries and in the biliary system has led to their introduction in urology. Use of metallic mesh stents in the ureter was first described in 1991 by Lugmayr and Pauer [5] and started a new era in relieving ureteric obstruction. Numerous reports of successful placement of mesh stents followed [6, 7], but they were used predominantly in patients who had extrinsic compression of the ureter by malignant disease and limited life expectancy [8, 9]. Since the turn of the century, authors have reported insertion of mesh stents for benign ureteric strictures in patients with expected long-term survival [2, 10, 11].

Placement of a metal mesh stent causes complications unique to the urinary system. Urine is highly lithogenic when it comes into contact with a foreign body (e.g., metal wire or nonabsorbable sutures). Even the early reports on use of mesh stents in the ureter describe incrustations with obstruction of the lumen after several months in patients with malignant disease and short life expectancy. Furthermore, urothelial hyperplasia and mucosal edema might cause short-term ureteral obstruction until the stent is fully incorporated under urothelial mucosa [5, 12]. Uncovered parts of stent wire serve as nidi for stone incrustations, as was observed in our patient with benign disease who had a mesh stent inserted for 15 years. Long-term hyperplasia of urothelium inside the lumen results in obstruction requiring double-J stent placement to reestablish patency [2, 6, 9, 11, 13] or even anecdotal removal of ingrown tissue with high-frequency rotablation [14]. Urinary obstruction in conjunction with the foreign material of the stent and incrustations are also risk factors for recurrent urinary tract infections, resulting in lumbar pain, hematuria, dysuria, and fever. To overcome the drawbacks of a nitinol mesh stent in the urinary tract, polytetrafluoroethylene-covered nitinol stents were developed and had promising short-term results [15]; however, distal migration of such stents poses a considerable problem [16].

Although procedures to restore patency of the mesh stent, including removal of incrustations, have been reported previously [17], to our knowledge, this paper is the first report of successful mesh stent removal from the ureter 15 years after insertion. In the literature, the number of patients with benign ureteric strictures that were treated by mesh stent insertion is much lower than the number of patients with advanced malignant disease who had limited life expectancy and had mesh stent inserted for a palliative cause. Furthermore, the design of the mesh stent promotes ingrowth of epithelium and submucosa between the wire struts and causes areas of fibrosis in submucosa, as observed in the canine model [18], preventing stent migration as well as its removal. There are two main reasons for the successful removal of the encrusted stent in our patient: (1) the stone incrustations that form on foreign materials (e.g., sutures and stents) in urine are relatively soft and were treated by multiple ESWL sessions before endourological procedures, and (2) the mesh stent was placed in the UPJ with the proximal part protruding into the renal pelvis, and that prevented the stent from becoming fully incorporated under the ureteric mucosa.

The sequence of procedures performed to remove the incrustations and mesh stent was dictated partially by patient's history and partially by the size of stone incrustations. Since in our patient percutaneous removal of mesh stent and incrustations was attempted already in 2007 and was unsuccessful and since percutaneous access would not allow visualization of the proximal ureter, where distal part of the mesh stent was incorporated, possibly risking complete ureteral tear, we have decided against PCNL as the first procedure. Also starting with URS and laser lithotripsy would be unsuccessful in clearing such a massive stone burden and also would not allow dislodging the mesh stent with intact large stone incrustations, which were fixing its position inside the renal pelvis. Moreover, limited maneuverability of the semirigid ureterorenoscope could not be overcome by usage of flexible ureterorenoscope, as sharp nitinol wires would damage the instrument. On the other hand, ESWL as starting procedure disintegrated substantial amount of incrustations, which were cleared through the nephrostomy tube, thereby aiding in dislodgement of the mesh stent during subsequent URS, which in turn allowed risk-free removal of dislodged mesh stent remnants by PCNL.

In conclusion, the use of combined minimally invasive methods for stone management should be considered, even when the long-term presence of foreign material makes treatment complicated, because an open surgical procedure in such a situation would result in nephrectomy in a vast majority of cases.

## Supplementary Material

Supplementary video file starts with a short video clip of ureterorenoscopy with laser lithotripsy of incrusted nitinol mesh stent, followed by a video clip of percutaneous nephrolithotomy procedure during which the remnants of incrusted mesh stent were removed from the kindey pelvis.
